# Surgery: To what extent can we operate?

**DOI:** 10.1002/ags3.12219

**Published:** 2018-10-25

**Authors:** Yoshihiro Kakeji

**Affiliations:** ^1^ Division of Gastrointestinal Surgery Department of Surgery Kobe University Graduate School of Medicine Kobe Japan

Surgery is one of the most effective treatments for cancer if the lesion is localized in a primary site or even in a metastatic one. Surgeons try to remove the tumor completely to make sure that there are no cancer cells left behind. The skill of surgeons has been continuously progressing with the assistance of endoscopic devices and robotic systems. Compared with human macroscopic vision and big hands, endoscopic magnified vision and tiny forceps or robotic articulated instruments have made it possible to carry out sophisticated operations. We can see small vessels and nerves clearly in the static view, and can separate the lesion from normal tissue without bleeding or nerve injury. Despite safe and accurate surgery, some advanced cancers will subsequently develop locoregional or distant metastases. The presence of occult nodal isolated tumor cells or micrometastases at the time of resection predisposes cancer patients to a high risk of disease recurrence. Adjuvant chemotherapy with newly developed agents is sometimes effective to prevent recurrence. Various protocols of post‐, pre‐, and perioperative adjuvant chemotherapy have verified efficacy thanks to clinical trials. Even for patients with distant metastasis which is difficult to resect, systemic chemotherapy sometimes makes it possible to carry out conversion surgery.

In this issue, Matsuyama and colleagues^1^ review the current state and future perspective of robotic‐assisted surgery for rectal cancer. Since April 2018, the public health insurance system in Japan has covered the cost of twelve robotic operations including rectal surgery. This provides a boost to increase robotic surgeries. Robotic articulated instruments allow surgeons to be extremely accurate during complex procedures, and to carry out well‐extended procedures, such as lateral lymph node dissection, dissection beyond the total mesorectal excision plane, or multivisceral resection. Using a robotic approach seems to develop the potential of surgeons both technically and oncologically, resulting in better patient outcome.

Kanaji et al^2^ reviewed recent updates in perioperative chemotherapy and recurrence patterns of gastric cancer. In Japan, postoperative adjuvant chemotherapy is the standard treatment strategy for pathological stage II and III gastric cancer. Fluoropyrimidine (S‐1) monotherapy reduced lymph node metastasis and peritoneal recurrence, whereas capecitabine and oxaliplatin reduced distant (liver, lung, distant lymph nodes, and other sites) metastasis. Intriguingly, S‐1 and docetaxel combination suppressed all types of recurrence including hematogenous, lymph node and peritoneal metastases. Effective neoadjuvant chemotherapy is a boon to patients who may suffer from postoperative chemotherapy. For patients with bulky nodal metastases, some clinical trials of neoadjuvant chemotherapy are now ongoing to evaluate promising protocols.

Pancreatic ductal adenocarcinoma (PDAC) remains a therapeutic challenge, as only 15%‐20% of all patients are candidates for upfront surgery at the time of diagnosis. This type of surgery offers the chance of long‐term survival. Hackert^3^ gives an overview on the current literature of treatment concepts in resectable, borderline resectable, or locally advanced cases. In locally advanced PDAC, neoadjuvant treatment approaches have recently resulted in high rates of secondary resection, thus allowing “conversion” surgery in an otherwise palliative treatment situation. Combined chemotherapy, FOLFIRINOX (leucovorin, fluorouracil, irinotecan, and oxaliplatin), seems to be the most effective possibility of achieving secondary resectability and consequently increasing the number of patients who can undergo pancreatectomy after a primary diagnosis of unresectability.

Surgery has become more effective to cure a patient with cancer in multimodal therapies. Resectability of cancer changes after chemo/chemoradiotherapy. However, there is still one clinical problem. It is sometimes difficult to diagnose the existence of viable cancer cells by present conventional imaging techniques after neoadjuvant treatment. To what extent should we resect the lesion by surgery? We have to continue to discuss this problem of how best to benefit patients before carrying out any surgical steps of no return.

## DISCLOSURE

Conflicts of Interest: Author declares no conflicts of interest for this article.
